# Statins for polycystic ovary syndrome in varying resource settings: a phenome-wide association study and evidence synthesis

**DOI:** 10.3389/fphar.2025.1562587

**Published:** 2025-08-13

**Authors:** Laura A. Zahn, Taylor D. Budine, Jana K. Shirey-Rice, Meghan M. Joly, Robert S. Wallis, Gordon R. Bernard, Kenneth J. Holroyd, Jill M. Pulley, Rebecca N. Jerome

**Affiliations:** ^1^ Vanderbilt Institute for Clinical and Translational Research, Vanderbilt University Medical Center, Nashville, TN, United States; ^2^ Aurum Institute, Johannesburg, South Africa; ^3^ Center for Technology Transfer and Commercialization, Vanderbilt University, Nashville, TN, United States

**Keywords:** simvastatin, statins, HMG-CoA reductase inhibitors, polycystic ovary syndrome, PheWAS, global health

## Abstract

**Background:**

There are many diseases prevalent around the globe that lack accessible and safe treatment options. Through Vanderbilt University Medical Center’s and Repurposing Essential Medicines Internationally program (Project Remedi), we aim to identify novel therapeutic uses for medications already approved and on the World Health Organization’s (WHO) Essential Medicines List (EML). We explored additional uses for simvastatin, an oral 3-hydroxy-3-methylglutaryl-coenzyme A (HMG-CoA) reductase inhibitor that is on the EML and may have additional therapeutic use outside of hypercholesterolemia.

**Methods:**

We conducted a phenome wide association study (PheWAS) of a 3-hydroxy-3-methylglutaryl-CoA reductase (HMGCR) gene single nucleotide polymorphism (SNP) Ile638Val in 35,000 patient samples to identify novel uses for simvastatin. We then assessed biologic rationale and existing clinical evidence base related to novel phenotypes for simvastatin use for key PheWAS results.

**Results:**

PheWAS of HMGCR variants identified a novel signal related to ovarian disease, in addition to a validating signal related to lipid dysfunction. Review of the literature substantiated involvement of HMG-CoA reductase signaling in hormone synthesis and posited involvement of dysfunction in this pathway in the development of polycystic ovary syndrome (PCOS). Synthesis of the literature regarding use of statins supported the role of these agents in improvement of symptoms and quality of life in women affected by PCOS who are not pregnant or trying to conceive, with a safety profile similar to this agent’s use in hyperlipidemia.

**Conclusion:**

Given the evidence supporting safety and efficacy of simvastatin for PCOS management, the widespread availability on the EML and affordability worldwide, simvastatin is a promising therapeutic avenue for PCOS. A large-scale efficacy trial would be valuable in further substantiating this use. Repurposing simvastatin, a widely available medicine, can provide clinicians and patients with an additional strategy for PCOS, especially in areas where medical care is limited.

## 1 Introduction

Timely and affordable access to effective and safe therapeutics is a key component of achieving meaningful improvements in global health equity. With that goal, the World Health Organization publishes a model Essential Medicine List (EML) updated every 2 years to serve as a guide for countries to prioritize what is included in their own national medicines policy (Laing et al., 2003). In low- and middle-income countries, in particular, medicines on the model list are more likely to be available in various countries, as EML designation can be used as a tool for negotiating medicine pricing and availability (Baxi et al., 2019).

While the EML plays an important role in making therapeutic strategies more widely available, it of course does not cover all conditions affecting humans. Thus, many significant diseases around the globe still may lack readily available treatment options. To help address this challenge, we began an initiative to identify new therapeutic uses for WHO-identified essential medicines called Project Repurposing Essential Medicines Internationally (Project Remedi). Project Remedi is a component of our academic institution’s drug repurposing program, which leverages genetic information, clinical data extracted from electronic health records, and evidence in the literature to explore novel indications for existing drugs and biological therapeutics (Challa et al., 2019; Pulley et al., 2017).

Project Remedi prioritizes therapeutic agents listed on the EML, and then leverages our repurposing methods to discover disease pathways targeted by those medicines (Challa et al., 2019; Challa et al., 2021; Pulley et al., 2020). This computational approach helps to uncover novel gene/disease associations that these pharmaceuticals can then be used to treat. Through this approach to uncovering new uses for affordable generic medications already on pharmacy shelves, it might be possible to make better use of available therapeutic strategies and increase the likelihood that patients will have access to the treatments they need.

Here, we report our application of these methods to simvastatin, an oral 3-hydroxy-3-methylglutaryl-coenzyme A (HMG-CoA) reductase inhibitor that has been widely and globally prescribed since the early 1990s for the treatment of hypercholesterolemia and reduction of cardiovascular morbidity and mortality in those at increased risk. It is currently listed on the WHO’s EML for the indications “mixed hyperlipidemia” and “coronary atherosclerosis”. Since simvastatin has a known mechanism of action related to a specific target (HMG-CoA) and is listed on more than 85 country-specific EMLs (Author anonymous, 2025a), ensuring relatively wide availability, we applied our repurposing approach to identify new potential indications for this therapeutic agent.

## 2 Materials and methods

We leveraged the strength of two key methods in exploring potential repurposing opportunities for simvastatin: 1) a phenome-wide association study (PheWAS) of a gene that plays an important role in several biological pathways and 2) comprehensive review of the literature to assess concordance with potential repurposing signals identified via PheWAS. Because the logic of PheWAS can be extended to predict phenotypic manifestations of pharmacological targeting (such as with simvastatin) of a given gene product in humans, we regularly use these methods for hypothesis generation related to drug repurposing (Challa et al., 2019; Pulley et al., 2017; Challa et al., 2021; Pulley et al., 2020).

### 2.1 PheWAS

We conducted a PheWAS explore disease associations with genetic variation in the 3-hydroxy-3-methylglutaryl-CoA reductase (HMGCR) gene. PheWAS is a well-established computational method that can identify diseases or conditions (phenotypes) that are associated with genetic variants within a target gene (Denny et al., 2010; Denny et al., 2013).

Our PheWAS analysis employed logistic regression, adjusted for sex and age as covariates, and included approximately 35,000 patients with available Illumina Exomechip genotyping data and phenotype data extracted from electronic health records at our large academic medical center. As we conceived of our PheWAS results as informing hypothesis generation and identification of a potential drug repurposing signal for further evaluation using the primary literature, we analyzed odds ratios as simple point estimates and did not implement a correction for multiple comparisons.

As simvastatin is an HMG CoA reductase inhibitor, acting to reduce cholesterol, we focused our PheWAS on variation within the HMGCR gene. Our Exomechip data for the HMGCR gene included a missense single nucleotide polymorphism (SNP), p. Ile638Val (chr5:75356374:A:G GRCh38; rs5908). A search of the literature did not identify any studies of the functional impact of this variant. The prevalence of this variant in a European non-Finnish population is estimated to be approximately 2% and *in silico* prediction tools (e.g., PolyPhen, CADD) predicted that this variant may not be deleterious (Author anonymous, 2025b). However, the location and nature of this variant suggests that it may be associated with impact on the protein structure and function. Amino acid changes can alter the tertiary structure of a protein, affecting folding and function in related downstream pathways. In the case of the HMGCR enzyme, the isoleucine to valine switch at position 638, which also resides in a known coding conserved region of the protein, could impact both the structure and stability of the protein as well as the ability to participate in protein-protein interactions (González-Castejón et al., 2011). This potential for functional impact served as our rationale for employing PheWAS to evaluate whether the putative effects on protein function were substantiated with phenotypes suggesting lipid-related effects *in vivo*.

### 2.2 Literature review

The studies from the literature for this analysis were identified by a trained information scientist searching the PubMed and Web of Science databases, as well as a broad Google search to identify unindexed and grey literature. The search was limited to English language publications and employed a range of search terms including: “polycystic ovary syndrome,” “PCOS” and “simvastatin”; a complementary search for “polycystic ovary syndrome” and (“atorvastatin” or “rosuvastatin” or “pravastatin” or “fluvastatin” or “statin” or “statins” or “hydroxymethylglutaryl-CoA” or “lovastatin” or “pitavastatin”) was also executed to add in adding to the context regarding potential class effects of statins in this disease. This search was not date limited and was last updated on 6 December 2024. The reference lists of articles were also reviewed, to identify any studies not found by the initial search and to better clarify preclinical and mechanistic underpinnings of both the disease and the therapy. No studies investigating the use of a statin for management of PCOS were excluded from this exploration and evidence from all identified reports was systemically extracted.

## 3 Results

### 3.1 PheWAS results: HMG-CoA reductase

In our PheWAS results (Table 1), we found a validation signal - increased risk of lipoprotein disorders - suggesting our candidate SNP p. Ile638Val is behaving *in vivo* as an HMG CoA reductase activator, thus having the opposite effect of simvastatin, which functions as an HMG CoA reductase inhibitor.

**TABLE 1:** HMGCR PheWAS results.

SNP (gene)	Phecode	Phenotype description	Cases (n)	Controls (n)	Odds ratio	P value	AFF_11*	AFF_12**
Validation signal								
rs5908 (HMGCR)	277.51	Lipoprotein disorders	23	24862	3.34000	0.0455	0	3
Novel signals								
rs5908 (HMGCR)	258.1	Postablative ovarian failure	39	11943	4.77000	0.0001169	0	7
rs5908 (HMGCR)	220	Benign neoplasm of ovary	87	10217	2.95100	0.00105	1	8

Key: HMGCR HMG-CoA, reductase gene; SNP, single nucleotide polymorphism; *AFF_11 homozygous carriers of the SNP, of interest; **AFF_12 heterozygous carriers of the SNP, of interest.

We also see a novel cluster of phenotypes indicating that this variant is associated with increased risk of ovarian dysfunction (Table 1). We next undertook an exploration of the evidence regarding involvement of lipid dysfunction and HMG-CoA reductase signaling in ovarian disease to assess whether a condition within this spectrum would present a repurposing opportunity for simvastatin.

#### 3.1.1 Evidence scan

##### 3.1.1.1 HMG-CoA reductase in ovarian pathophysiology and PCOS

**FIGURE 1:**
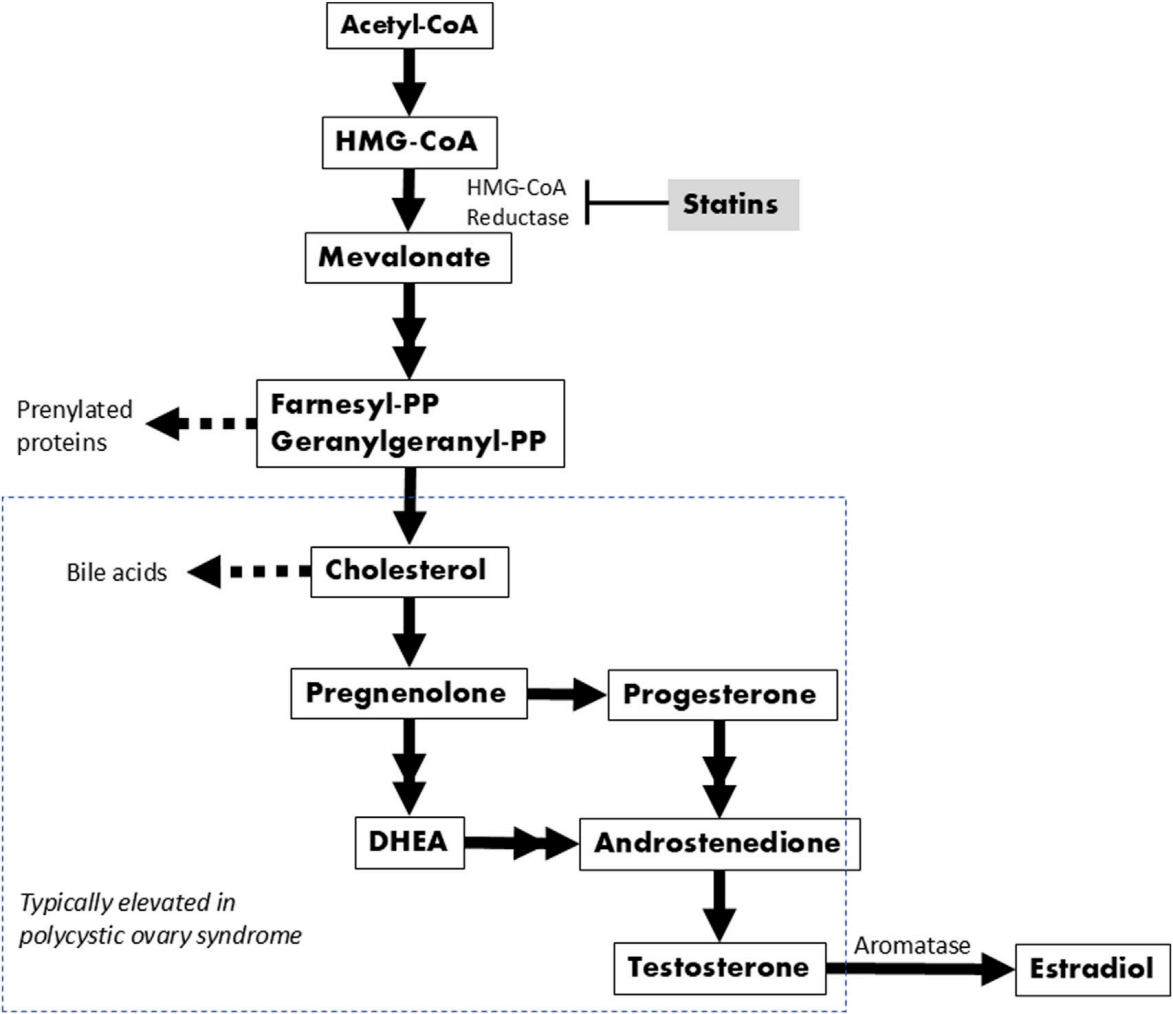
Biochemical pathway leading to cholesterol and sex hormone synthesis. Double arrows indicate where one or two intermediates were omitted.

HMG-CoA reductase is the rate-controlling enzyme of cholesterol biosynthesis and is responsible for converting HMG-CoA to mevalonate, a precursor for a number of important molecules in addition to cholesterol (Figure 1). Thus, the connection between this enzyme and the ovaries is not surprising when considering that cholesterol is required for the synthesis of steroid hormones, including estrogens and androgens like testosterone (Smals et al., 1991; Eacker et al., 2008). Within a healthy ovary, these hormones are precisely regulated ensuring mono-follicular ovulation during the menstrual cycle. However, in the setting of excess androgens, as is likely the case for this SNP, there is an increased density of preantral follicles which then prematurely arrest at the antral phase before ovulation can occur (Franks and Hardy, 2018). Rather than releasing their contents and regressing, the arrested follicles stay intact within the ovaries, resulting in fluid filled cysts--a hallmark of PCOS. Furthermore, cholesterol and its downstream components are often elevated in PCOS patients, independent of body mass index (Wild et al., 2011; Liu et al., 2019; Yang et al., 2021). As a HMG-CoA reductase inhibitor, simvastatin interrupts this pathway early by inhibiting the enzyme HMG-CoA reductase, limiting the conversion of HMG-CoA to mevalonate and decreasing the amount available for both cholesterol and androgen synthesis. The enzyme aromatase is responsible for converting testosterone into estradiol, but several studies have shown reduced aromatase activity in PCOS patients (Ashraf et al., 2019). Thus, while PCOS can result from a variety of causes independent of HMGCR, our PheWAS results support the idea that alterations in the HMGCR/mevalonate pathway could be one promising target for treating the manifestations of this condition.

While the precise cause of PCOS is not well understood, it is thought to be multifactorial (Melo et al., 2015), with abnormalities in the steroid synthesis pathway commonly implicated, including alterations in the enzyme aromatase responsible for catalyzing the conversion of androgens into estrogens (Ashraf et al., 2019). It is also the most common endocrinopathy affecting reproductive-aged women in the world, with a global age-standardized prevalence rate of 4%–20% (Deswal et al., 2020). Generally, it presents as a spectrum of heterogeneous disorders of reproduction and metabolism in women with frequent symptoms such as: abnormal menstruation, infertility, obesity, hirsutism, acanthosis nigricans, acne, alopecia, and ovarian cysts. PCOS is a leading cause of infertility, which is a global public health issue. (WHO, 2025) Although PCOS is not life-threatening, women with PCOS have a substantially reduced quality of life (Taylor and Francis, 2020). Obesity and visible signs of excess androgens have prominent effects on physical appearance that can subsequently affect neuropsychological status, including increased risk of depression (Podfigurna-Stopa et al., 2015). Further, women with PCOS are at a higher risk of a range of significant health conditions including impaired glucose tolerance, type II diabetes, cardiovascular disease, hypertension, and metabolic syndrome (Khan et al., 2019).

Currently, there is no cure identified for PCOS, only symptomatic treatments. One of the first lines of defense is combined oral contraceptives to mitigate the symptoms of PCOS by balancing hormones and restoring menstrual regularity, improving hyperandrogenism, and reducing unwanted hair growth (Oguz and Yildiz, 2021; de Melo et al., 2017). However, oral contraceptives have adverse effects that have potentially concerning overlap with known comorbidities of PCOS, such as the heightened risk of venous thromboembolism and other cardiovascular events (Oguz and Yildiz, 2021). Additional strategies are also used in PCOS for management of its manifestations, including diet and lifestyle modification and pharmacologic and surgical interventions for obesity and metabolic dysfunctions such as diabetes (Wang et al., 2018).

### 3.2 Evidence scan: statins in PCOS

After finding substantive evidence supporting a connection between HMG-CoA reductase and PCOS, we next assessed the evidence describing the safety and efficacy of simvastatin and other statin class drugs in treatment PCOS. As described below in more detail, from a pool of approximately 300 items retrieved by our search, we identified a body of trial literature (n = 15), observational research (n = 3), and systematic reviews (n = 8) that indicated benefits associated with simvastatin therapy in women affected by PCOS, including positive effects on lipid levels and other measures of disease activity including hormone levels, body mass index (BMI), acne, and hirsutism.

#### 3.2.1 Systematic reviews and meta-analyses

We identified eight systematic reviews and meta-analyses that included evaluations of various statins, including simvastatin, atorvastatin, and rosuvastatin, for the management of PCOS, ranging in publication date from 2012 to 2023 (Gao et al., 2012; Ca et al., 2016; Yang et al., 2019; Almalki et al., 2020; Chen et al., 2021; Miao and Zhou, 2022; Hao et al., 2023; Xiong et al., 2023) (Table 2). There is partial overlap among studies represented in these systematic reviews; however, variation in inclusion criteria (typically including any statin), search approach, analytic techniques, and outcomes of interest resulted in variability in their detailed conclusions regarding utility of simvastatin and other statins in this condition.

**TABLE 2:** Systematic reviews and meta-analyses including simvastatin, atorvastatin, and/or rosuvastatin in polycystic ovary syndrome.

*Author, year*	*Design*	*Statin tested*	*Total N*	*Finding(s)*
Hao et al. (2023)	Systematic review and network meta-analysis of 13 RCTs; 7 trials including a statin	Atorvastatin was tested in five trials; simvastatin plus metformin was tested in two trials; and simvastatin monotherapy was tested in one trial	370 in studies involving a statin	Atorvastatin (WMD -3.1, 95% CrI: 3.7 to −2.5), metformin + simvastatin (WMD -2.8, 95% CrI: 4.1 to −1.5), simvastatin (WMD -2.7, 95% CrI: 4.2 to −1.3), were each significantly more effective in reducing total T vs placebo (P < 0.05) No between-group significant difference observed in incidence of study withdrawal due to adverse events
Xiong et al. (2023)	Systematic review of 6 RCTs	3 trials evaluated atorvastatin and 3 trials evaluated simvastatin	396	Conclusions from 3 trials comparing statin to placebo: uncertain whether statin shorted mean length of menstrual cycle (−2 days, 95% CI -24.86 to 20.86) or testosterone (mean difference at 6 weeks 0.06, 95% CI -0.72 to 0.84; similar uncertainty at 3 and 6 months) Conclusions from trial comparing statin plus metformin to metformin alone: uncertain if statin affected menstrual regularity (MD 0.60 menses, 95% CI 0.08–1.12), hirsuitism (MD -0.16, 95% CI -0.91 to 0.59), acne severity (MD -0.31, 95% CI -0.67 to 0.05) or testosterone (MD -0.03, 95% CI -0.37 to 0.31) Conclusion from two trials comparing statin to metformin: uncertain whether statin improved frequency of menses (MD 0.50 menses, 95% CI -0.05–1.05) reduced hirsutism (MD -0.26, 95% CI -0.97 to 0.45), reduced acne severity (MD -0.18, 95% CI -0.53 to 0.17), or reduced testosterone levels (MD -0.24, 95% CI -0.58 to 0.10) Conclusions from trial comparing stain plus OCP to OCP alone: uncertain if statin affected menstrual regularity (MD -0.12, 95% CI -0.41 to 0.17) or testosterone (MD -0.82, 95% CI -1.38 to −0.26) No significant adverse events noted Evidence for each category rated as very low certainty
Miao and Zhou (2022)	Meta-analysis of 13 RCTs	Simvastatin (7) Atorvastatin (5) Rosuvastatin (1)	869	Significant reduction in total T, free T, DHEAs, androstenedione, LH, LH to FSH ratio, and prolactin; Significant reduction in total cholesterol; LDL, and triglycerides; Significant reduction fasting glucose, insulin sensitivity index, and high-sensitivity CRP
Chen et al. (2021)	Meta-analysis of 9 RCTs	Simvastatin (6) Atorvastatin (3)	682	All studies found significantly reduced T in association with statin treatment; reduced DHEA found in 7 studies
Almalki et al. (2020)	Meta-analysis of 9 RCTs, including 4 that involved a statin	Different types of statin at any dose; 2 trials included simvastatin and 2 included atorvastatin	238 in studies involving a statin	In the network meta-analysis, atorvastatin showed greater reduction in T compared to COC (MD −2.78, 95%CrI −3.60, −1.97), spironolactone plus metformin (MD −2.83, 95%CrI −3.80, −1.87), simvastatin (MD −2.88, 95%CrI −3.85, −1.92), spironolactone (MD −2.90, 95%CI −3.77, −2.02), simvastatin plus metformin (MD −2.93, 95%CrI −3.79, −2.06), metformin (MD −2.97, 95%CrI −3.69, −2.25), lifestyle modification (MD −3.02, 95%CrI −3.87, −2.18), and placebo (MD −3.04, 95%CrI −3.56, −2.53)
Yang et al. (2019)	Meta-analysis of 10 RCTs	Different types of statin at any dose that continued for at least 2 weeks Six studies simvastatin (20 mg/day), one study atorvastatin (40 mg/day), one study rosuvastatin (10 mg/day)	735	Atorvastatin significantly reduced DHEA (SMD, −0.63; 95% CI, −1,20 to −0.05; p = 0.03; I2 = 38%) but simvastatin did not significantly reduce DHEA (SMD: −0.14; 95% CI, −0.49–0.28; p = 0.43; I2 = 77%) Subgroup analysis based on duration of treatment showed no significant difference between 12 weeks (SMD, −0.61; 95% CI, −1.23–0.02; p = 0.06; I2 = 85%) and 24 weeks (SMD, −0.34; 95% CI −0.95–0.28; p = 0.29; I2 = 83%) of statin treatment
Ca et al. (2016)	Systematic review of 12 trials	Trials involved simvastatin or atorvastatin (11 trials); one study evaluated rosuvastatin. Most trials were 12 weeks duration; a low to moderate dose of statin was typically used	NR	10 of 12 trials showed reduction in testosterone levels or other hormones (DHEA-S and androstenedione); some trials showed improvement in LH/FSH ratio; all trials demonstrated positive effects on lipid profiles No synthesis of overall results by statin type within this review
Gao et al. (2012)	Meta-analysis of 4 RCTs	Simvastatin (2 trials) Atorvastatin (2 trials) Regimen details included: atorvastatin 40 mg, QD vs. placebo 6 weeks; simvastatin 20 mg, QD vs. metformin 850 mg, BID vs. sim + met 20mg, QD + 850 mg, BID 12 weeks simvastatin 20 mg, QD vs. placebo 12 weeks atorvastatin 20 mg, QD vs. placebo 12 weeks	254	Significant reduction of serum total testosterone when comparing statin with placebo (Std MD = − 3.03, 95%CI − 5.85 ∼ − 0.22, P = 0.03) or statin + metformin with metformin (Std MD = − 1.07, 95%CI: −2.06∼ − 0.07, P = 0.04). Statin was more effective than placebo in reducing LDL (WMD = − 0.87, 95%CI − 1.18∼ − 0.55, P < 0.0001), TC (WMD = − 1.23 95%CI − 1.35∼ − 1.11, P < 0.00001), TG (WMD = − 0.50, 95%CI − 0.73∼ − 0.27, P < 0.00001); and statin + metformin was more effective than metformin in lowering LDL (WMD = − 0.84, 95%CI:− 1.33 ∼ − 0.354, P = 0.0009), TC (WMD = − 1.28, 95%CI: −1.47 ∼ − 1.10, P < 0.00001), and TG (WMD = − 0.27, 95%CI:− 0.36∼ − 0.19, P < 0.00001)

Key: BID, twice a day; BMI, body mass index; COC, combined oral contraceptives; CrI, credible interval; CRP, C-reactive protein; DHEA, dehydroepiandrosterone; DHEA-S, dehydroepiandrosterone sulfate; LDL, low density lipoprotein; LH, luteinizing hormone; FSH, follicle stimulating hormone; MD, mean difference; OCP, oral contraceptive pill; QD, once a day; RCT, randomized controlled trial; SMD, standardized mean difference; T, testosterone; TC, total cholesterol; TG, triglycerides; WMD, weighted mean difference.

Five of these systematic reviews focused on evaluation of statins as a class of therapeutics (Gao et al., 2012; Ca et al., 2016; Chen et al., 2021; Miao and Zhou, 2022). Two included comparison of different statins including simvastatin, atorvastatin, and/or rosuvastatin (Yang et al., 2019; Almalki et al., 2020). Most analyses found improvements in hormone levels (e.g., reductions in total testosterone, free testosterone, and dehydroepiandrosterone/DHEA) and lipid profiles (e.g., total cholesterol, LDL, and triglycerides) associated with the use of at least one of the statins. None of the reviews identified notable adverse effect signals. The two meta-analyses comparing different statins found atorvastatin to be superior to the other statins in terms of effects on testosterone (Almalki et al., 2020) or DHEA (30), although the small size of the comparative data pool may limit the clinical utility of these findings. Given the heterogeneity in the clinical trials and the relatively small sample sizes, however, the overall certainty of the evidence was rated very low, indicating that the results should be interpreted with caution. Despite the low certainty, seven of these reviews concluded that statin therapy is a reasonable option for women affected by PCOS and recommended further research to evaluate long-term effects and optimal dosing (Gao et al., 2012; Ca et al., 2016; Yang et al., 2019; Almalki et al., 2020; Chen et al., 2021; Miao and Zhou, 2022; Hao et al., 2023). The final systematic review concluded that the low certainty in available evidence precluded conclusions about efficacy (Xiong et al., 2023).

#### 3.2.2 Randomized controlled trials (RCTs)

Fifteen randomized controlled trials, briefly summarized and cited below, compared the utility of simvastatin-containing regimens to one or two other treatment options. Small trial sizes predominated and trials were heterogeneous in approach, duration, and biochemical outcome measures. Trial data generally indicated positive effects of simvastatin therapy on lipids, hormone levels, and other measures of disease activity in women with PCOS. The range of comparisons in this literature include.

##### 3.2.2.1 Simvastatin to placebo

There are two trials comparing simvastatin to placebo, one focusing on women with PCOS pursuing *in vitro* fertilization (Rashidi et al., 2011) and one on women with general PCOS(37). The IVF in PCOS trial found positive effects on testosterone and cholesterol, but did not find benefit in terms of IVF success (Rashidi et al., 2011). The PCOS trial supported the positive effects of simvastatin as compared with placebo on a range of outcomes including hormones (e.g., testosterone, DHEA), lipids, menstrual regulatory, hirsutism, acne, ovarian volume, body mass index, and waist hip ratio; this trial did not find benefit of simvastatin therapy on fasting glucose, fasting insulin, or HOMA-IR index (insulin resistance measure). (Seyam et al., 2018).

##### 3.2.2.2 Simvastatin and metformin

There are multiple trials evaluating simvastatin effectiveness in comparison to metformin, a well-known diabetes medication. Three trials compared simvastatin to metformin in PCOS (Navali et al., 2011; Pourmatroud et al., 2015). One trial focused on PCOS and found superiority of simvastatin in effects on lipids, CRP, and acne; metformin performed better in terms of effects on blood sugar and insulin measures (Navali et al., 2011). The second trial focused on women with PCOS pursuing *in vitro* fertilization (IVF); both regimens were associated with beneficial effects on biochemical parameters, but neither regimen had an effect on IVF outcomes (Pourmatroud et al., 2015). Additionally, three trials compared simvastatin to metformin to simvastatin plus metformin (i.e., three-arm trials; one trial reported in two papers) (Seyam and Hefzy, 2018; Karakas et al., 2013; Banaszewska et al., 2009; Banaszewska et al., 2011). All three found superiority in the simvastatin-containing arms compared to metformin alone in terms of effects on lipids, hormone levels (e.g., testosterone), and other measures of disease activity. Similarly, two trials evaluated simvastatin plus metformin to metformin alone (Malik et al., 2018; Kazerooni et al., 2010). Both trials found superiority of the simvastatin-containing arm related to improvements in hormone levels (e.g., testosterone, luteinizing hormone, follicle stimulating hormone) and in lipids.

##### 3.2.2.3 Simvastatin compared with other therapeutics

Finally, RCTs containing other therapeutics were compared to simvastatin treatments. One trial compared simvastatin to metformin to flutamide plus oral contraceptives (a three-arm trial) (Mehrabian et al., 2016), finding that simvastatin was superior to the other two regimens in terms of effects on waist circumference, BMI, and triglyceride levels. Metformin was superior in effects on fasting blood sugar. Another two trials compared simvastatin to atorvastatin (Kaya et al., 2010; Kaya et al., 2009). Both trials found the statin regimens lead to improvements in lipid levels and other measures of disease activity, while benefits attributed to the individual agents varied to some extent; the data suggests possible greater effects of simvastatin on hormone levels, while atorvastatin may have a greater impact on measures of insulin resistance. Similarly, two trials evaluated simvastatin plus oral contraceptives (OCPs) to OCPs alone (Duleba et al., 2006). Both studies found significant benefit in the combined therapy group, attributed to the addition of simvastatin, including improvement in hormone levels (testosterone, FSH, LH), lipids, and other measures of disease activity (e.g., hirsutism). An additive effect of simvastatin therapy on measures related to blood glucose or insulin was not identified in either trial.

##### 3.2.2.4 Observational data

In complement to the RCT data, we identified three observational studies (Table 3) which also indicated benefit of simvastatin in managing lipids and other parameters in small groups of women affected by PCOS (Azargoon et al., 2013; Krysiak et al., 2014; Yang et al., 2016). Two of these reports focused on simvastatin only (Azargoon et al., 2013; Yang et al., 2016); one study was a prospective cohort comparing simvastatin to ezetimibe (Krysiak et al., 2014).

**TABLE 3:** Clinical studies evaluating simvastatin-containing regimens in polycystic ovary syndrome.

*Author, year*	*Design*	*Statin or comparator*	*Total N*	*Finding(s)*
Trials comparing simvastatin to placebo				
Seyam et al. (2018)	Double-blind RCT	Simvastatin (20 mg/day) OR placebo for 6 months	200	Reduced T, LH/FSH ratio, total cholesterol, LDL, triglycerides, DHEAS and FSH. Increased HDL. Improved menstrual regularity. Decreased hirsutism, acne, ovarian volume, BMI and waist/hip ratio. No adverse events
Rashidi et al. (2011)	Double-blind RCT	Simvastatin (20 mg/day) OR placebo for 8 weeks	61	Reduced total T, total serum cholesterol and LDL but increased systemic inflammatory markers. No AEs
Simvastatin trials including use of metformin, oral contraceptives, and/or flutamide				
Seyam and Hefzy (2018)	Double-blind RCT	Simvastatin (20 mg/day) OR metformin (500 mg/day) OR simvastatin + metformin for 12 months	200	Improved spontaneous menses per 6 months, ovulation, volume of both ovaries, waist/hip ratio, hirsutism score, acne, total T, free T, DHEAS, SHBG, LH/FSH ratio, prolactin, total cholesterol, LDL, HDL and triglycerides. No AEs
Malik et al. (2018)	RCT	Simvastatin plus metformin OR metformin only for over 3 months	108	Improved LH/FSH ratio
Mehrabian et al. (2016)	Single-blind clinical trial	Simvastatin (20 mg/day) OR metformin OR flutamide + low dose oral contraceptives for 6 months	102	Reduced waist circumference, triglycerides, fasting blood glucose, CRP and BMI. No AEs
Pourmatroud et al. (2015)	Double-blind RCT	Simvastatin (20 mg/day) OR metformin administered for 8 weeks before starting ICSI cycle (both groups on daily low dose oral contraceptive pills for 8 weeks)	40	Reduced hirsutism, T, FSH, LH, total cholesterol, and LDL. Increased HDL. No AEs
Karakas et al. (2013)	RCT	Simvastatin (20 mg/day) OR metformin OR simvastatin + metformin for 3 months	62	Decreased total cholesterol, LDL, CRP, triglycerides and fasting insulin. Increased insulin sensitivity. No AEs
Navali et al. (2011)	Double-blind RCT	Simvastatin 20 mg/day OR metformin 500 mg 3x day for 3 months	400	Decreased percent of abnormal periods, acne, positive CRP, hyperinsulinemia, mean serum total cholesterol, LDL and DHEAS. Mean serum HDL increased. No side effects
(Banaszewska et al., 2011; Banaszewska et al., 2009)	RCT	Simvastatin (20 mg daily) OR metformin OR simvastatin (20 mg daily) plus metformin (850 mg twice daily) for 6 months	97	Simvastatin was superior to metformin in improving markers of systemic inflammation and endothelial function (i.e., reduction in hs-CRP and sVCAM)
Kazerooni et al. (2010)	Double-blind RCT	Simvastatin (20 mg/day) plus metformin OR metformin plus placebo for 12 weeks	84	Significantly reduced serum T levels, DHEAS, LH, LH/FSH ratio, total cholesterol, LDL and triglycerides and increased HDL.
Banaszewska et al. (2007)	Randomized crossover trial	Simvastatin (20 mg/day) + oral contraceptive pill for 12 weeks followed by oral contraceptive pills only for an additional 12 weeks, or *vice versa*	48	Reduced total T, free T, LH, LH/FSH, hirsutism, total cholesterol, LDL, hs-CRP and sVCAM-1. No side effects
Duleba et al. (2006)	RCT	Simvastatin (20 mg/day) + oral contraceptive pill OR oral contraceptive pill only for 12 weeks	48	Reduced serum T, DHEAS, LH, LH/FSH, total cholesterol, LDL and hirsutism, and increased HDL. No side effects
Trials comparing simvastatin to atorvastatin				
Kaya et al. (2010)	RCT	Simvastatin (20 mg/day) OR atorvastatin (20 mg/day) for 3 months	64	Both groups reduced total cholesterol and HDL-C. Simvastatin significantly reduced LH, FSHFAI, MDA, total T and hirsutism. Atorvastatin significantly reduced CRP, HOMA index, fasting insulin and LDL. No AEs
Kaya et al. (2009)	RCT	Simvastatin (20 mg/day) OR atorvastatin (20 mg/day) for 12 weeks	52	Both drugs reduced serum homocysteine levels, free T and total T. Both groups increased B-12. Homeostatic model assessment index was significantly lower in atorvastatin. No AEs
Observational studies of simvastatin				
Yang et al. (2016)	Prospective parallel groups matched by age	Simvastatin (20 mg/day) for 6 months compared to pre-treatment and healthy controls	42	Reduced cholesterol, triglyceride, LDL, HOMA-IR and markers of endothelial dysfunction
Azargoon et al. (2013)	Quasi experimental study (no comparator)	Simvastatin (20 mg/day) for 2 months (no comparator)	25	No significant change in BMI. No side effects
Quasi-experimental comparison of simvastatin to ezetimibe				
Krysiak et al. (2014)	Prospective parallel groups matched by age and weight	Simvastatin (40 mg/day) OR ezetimibe for 90 days	28	Significantly reduced total cholesterol, LDL, serum T, free T, androstenedione, and DHEAS. Modestly reduced LH/FSH ratio. No AEs

Key: AEs, adverse events; BMI, body mass index; CRP, c-reactive protein; DHEAS, dehydroepiandrosterone sulfate; FSH, follicle stimulating hormone; HDL, high density lipoprotein; HOMA-IR, homeostatic model assessment of insulin resistance; LDL, low density lipoprotein; LH, luteinizing hormone; RCT, randomized controlled trial; SHBG, sex hormone-binding globulin; sVCAM-1, soluble vascular cell adhesion molecule-1; T, testosterone.

#### 3.2.3 Safety considerations

Review of the literature describing use of simvastatin or atorvastatin in treatment of women with PCOS indicates a safety profile comparable to that observed in the substantive evidence base on statin use in hyperlipidemia. A detailed extraction of these concordant safety findings from all identified studies on use of simvastatin (Rashidi et al., 2011; Seyam et al., 2018; Navali et al., 2011; Pourmatroud et al., 2015; Seyam and Hefzy, 2018; Karakas et al., 2013; Banaszewska et al., 2009; Banaszewska et al., 2011; Malik et al., 2018; Kazerooni et al., 2010; Mehrabian et al., 2016; Kaya et al., 2010; Kaya et al., 2009; Duleba et al., 2006; Azargoon et al., 2013; Krysiak et al., 2014; Yang et al., 2016; Banaszewska et al., 2007) are outlined in Table 3; similar data regarding safety findings with use of atorvastatin in PCOS(54–64) and rosuvastatin (Ghazeeri et al., 2015; Celik and Acbay, 2012) are also included for context (Table 4). Regarding possible side effects of simvastatin, there has been an association between statin use and dose dependent skeletal muscle damage including rare risk of rhabdomyolysis (Di Stasi et al., 2010; Skottheim et al., 2008; Thompson et al., 2003), although there is new evidence suggesting that overall these changes are mild and clinically insignificant in most patients (Re et al., 2022). Clinicians can use judgment on an individual case basis to determine if the benefits outweigh the risks of myalgia.

**TABLE 4:** Clinical studies evaluating other statin-containing regimens in polycystic ovary syndrome (atorvastatin, rosuvastatin).

*Author, year*	*Design*	*Statin or comparator*	*Total N*	*Finding(s)*
Atorvastatin trials				
Shawish et al. (2021)	Meta-analysis	Atorvastatin (6)	6 RCTs 265	Reduced total T, FAI, androstenedione and DHEAS
Chen and Zheng (2021)	Meta-analysis	Atorvastatin (9)	9 RCTs 406	Decreased fasting insulin; Lower HOMA-IR value; No significant effect on fasting glucose or BMI
(Sathyapalan et al., 2009; Sathyapalan et al., 2010a; Sathyapalan et al., 2010b; Sathyapalan et al., 2012a; Sathyapalan et al., 2012b; Sathyapalan et al., 2017; Sathyapalan et al., 2019)	RCT	Atorvastatin (20 mg/day) or placebo for 3 months	40	Reduced total cholesterol, LDL cholesterol, triglycerides, CRP, free androgen index, testosterone and insulin resistance per HOMA-IR. Atorvastatin increased SHBG.
(Puurunen et al., 2013; Luotola et al., 2018)	RCT	Atorvastatin (20 mg/day) or non-statin placebo for 6 months	28	Reduced serum levels of DHEA, CRP, total cholesterol, LDL cholesterol, triglycerides. No change in serum T
Krysiak and Okopien (2014)	RCT	Atorvastatin (40 mg daily) or atorvastatin (20 mg daily) plus ezetimibe (10 mg daily)	23	Both dosages decreased total and LDL cholesterol. High-dose reduced total T, free T and androstenedione
Raja-Khan et al. (2011)	RCT	Atorvastatin (40 mg) or placebo once daily for 6 weeks	20	Reduced systolic and diastolic blood pressure, LDL cholesterol, triglycerides, androstenedione and DHEAS.
Rosuvastatin trials				
Ghazeeri et al. (2015)	Double-blind RCT	All patients received rosuvastatin 10 mg/day for 3 months after starting the study Then randomized to either rosuvastatin (10 mg/day) + metformin (850 mg twice daily after meals) or rosuvastatin (10 mg/day) + placebo	37	Reduced cholesterol
Celik and Acbay (2012)	RCT	Rosuvastatin (10 mg/day) + lifestyle changes + metformin or lifestyle changes + metformin only	38	Reduced hsCRP, triglyceride, total cholesterol, and LDL cholesterol

Key: BMI, body mass index; CRP, c-reactive protein; DHEA, dehydroepiandrosterone; DHEAS, dehydroepiandrosterone sulfate; FAI, free androgen index; HOMA-IR, homostatic model assessment of insulin resistance; hsCRP, high-sensitivity c-reactive protein; LDL, low density lipoprotein; RCT, randomized controlled trial; SHBG, sex hormone-binding globulin; T, testosterone.

##### 3.2.3.1 Caution regarding risks to fetus

Of particular import within the PCOS population, women who are pregnant or attempting to conceive should not use a statin due to risk of fetal harm and miscarriage (Vahedian-Azimi et al., 2021; Zarek and Koren, 2014).

#### 3.2.4 Dosing and treatment approach

The studies describing use of simvastatin in PCOS typically employed an orally administered dose of 20 mg per day, within the range used to treat hyperlipidemia (ZOCOR, 1991). While doses of up to 40–80 mg per day are employed in hyperlipidemia, only one study identified in the current evidence synthesis used a simvastatin dose greater than 20 mg day; this small prospective observational study used a dose of 40 mg/day and observed outcomes similar to the studies that employed the 20 mg/day dose (Krysiak et al., 2014). Monitoring of lipids and other metabolic parameters (e.g., blood glucose, liver function tests) should be pursued when clinically indicated, as per usual care in this condition.

As noted above, simvastatin should *not* be used in women who are pregnant or attempting to conceive, due to risk of fetal harm and miscarriage.

## 4 Discussion

### 4.1 Implications of PheWAS results

Our PheWAS results, combined with the literature evidence summarized here supporting its safety and efficacy in PCOS, suggest that the use of simvastatin could be expanded to include treatment of patients affected by polycystic ovary syndrome, although a large-scale clinical trial would be valuable in confirming this. Given its safety profile and the lack of other therapies, we propose that simvastatin is a potentially viable treatment option for patients suffering with PCOS. However, simvastatin is contraindicated in women who are pregnant or attempting to conceive, due to associated risk for the fetus. It will be important to monitor the supporting evidence base, particularly the trial literature, for guiding strength of evidence and recommendations for statins in PCOS as the literature evolves.

### 4.2 Evidence for simvastatin in PCOS

An excess in androgen levels is responsible for many of the physical symptoms of PCOS, including hirsutism, acne, and hair loss, and the cumulative evidence presented here indicates statin therapy can significantly reduce both free and total testosterone levels as well as DHEAS and androstenedione (Rashidi et al., 2011; Seyam et al., 2018; Pourmatroud et al., 2015; Seyam and Hefzy, 2018; Kazerooni et al., 2010; Kaya et al., 2010; Kaya et al., 2009; Duleba et al., 2006; Krysiak et al., 2014; Banaszewska et al., 2007). While a reduction in hormone levels may not always translate to a reduction in symptoms, most studies that reported such outcomes included positive results, most commonly reduced hirsutism, but also notable improvements in acne, menstrual regularity, and obesity measures (Seyam et al., 2018; Navali et al., 2011; Pourmatroud et al., 2015; Mehrabian et al., 2016; Kaya et al., 2010; Duleba et al., 2006; Seyam and Hefzy, 2018). Dyslipidemia is a common complication in PCOS patients, affecting an estimated 70% and usually manifesting as an elevation in LDL, total cholesterol, and triglycerides and a reduction in HDL levels, correlating with increased risk in cardiovascular disease (Legro et al., 2001; Liu et al., 2019). As statins are well known to reduce levels of harmful lipids by interfering with cholesterol production, it is reassuring that many studies of simvastatin use in PCOS patients also reported significant improvements in lipid profiles, an effect that is generally beneficial for overall health outcomes (Rashidi et al., 2011; Seyam et al., 2018; Navali et al., 2011; Pourmatroud et al., 2015; Karakas et al., 2013; Mehrabian et al., 2016; Kaya et al., 2010; Kazerooni et al., 2010; Duleba et al., 2006; Krysiak et al., 2014; Yang et al., 2016; Banaszewska et al., 2007; Seyam and Hefzy, 2018). The polycystic nature and size of the ovaries in these patients can only be identified via ultrasonography, with transvaginal ultrasound the most reliable, but most statin studies published so far did not assess the effects on ovarian morphology. The only exceptions are the two RCT’s published by Seyam et al., in 2018, which both determined a decrease in ovarian volume after statin treatment (Seyam et al., 2018; Seyam and Hefzy, 2018). Given that large and/or numerous ovarian cysts can induce significant pelvic pain and impact fertility, any reductions in ovarian volume are presumably beneficial for a patient’s quality of life.

### 4.3 Global health context and accessibility

The WHO has identified the health of women and girls a particular concern given that sociocultural factors can impact the quality and timeliness of healthcare services they have access to (World Health Organization, 2035). Most estimates suggest 70% of the world’s poor are female, and this income disadvantage places a higher burden on female health due to malnutrition, greater exposure to environmental health risks, and reduced access to care (Sicchia and Maclean, 2006). The WHO’s model EML is one strategy attempting to increase access to the most beneficial and cost-effective medications for those in need. For example, treatments for PCOS can improve overall health and the quality of life for women worldwide. The presence of statins on the EML points to the fact that they are more available in countries with various resource levels. Although there is some evidence supporting the use of simvastatin as a treatment response to PCOS and evidence that simvastatin can improve biochemical markers in this condition, the burden of evidence has not yet risen to the threshold required by the EML and providers considering use of statins in PCOS will continue to evaluate potential risk and benefit on a case by case basis in the absence of practice guidelines. Repurposing widely available medications so that they can be reliably used in multiple conditions is one way to tackle key global health challenges in a resource-sensitive manner and we continue to seek these opportunities.

### 4.4 Limitations

Our PheWAS findings are based on data from a single institution and leverage phenotypic effects of a variant with limited functional information; it would be premature to consider this variant to be a pharmacogenomic signal, and rather presents data on the hypothesis generation end of the research continuum. Our approach also employed PheWAS as a hypothesis generation mechanism and did not use a multiple correction threshold, relying on the primary literature for further triangulation regarding whether statins may play a role in treatment of PCOS. While the lipid-related phenotypes revealed by PheWAS suggest that this variant has impact and the clinical trial literature indicates a role for statin therapy in PCOS, it will be important to replicate this work in larger datasets. Given that the clinical trial literature is relatively small and heterogeneous, additional studies to confirm these associations and further elucidate the role that statin therapy can play within the therapeutic landscape for PCOS will be an important endeavor for future research.

## 5 Conclusion

Our review evaluated evidence for simvastatin in PCOS management. Our findings suggest simvastatin has potential as an affordable, globally accessible treatment for PCOS with promising effects on lipid profiles, hormone regulation, and symptom improvement. However, we highlight the need for further research, including large-scale trials, to address evidence gaps and resource-specific challenges for global applicability. We anticipate that novel methods such as our Project Remedi, which employs cutting edge technology for scientific breakthroughs in drug repurposing to identify new therapeutic targets for medications commonly used within the EML, will inform repurposing strategies in the future and further enrich the global treatment landscape for unmet medical needs.

## Data Availability

The dataset used to generate the results presented in this article is not readily available because data are derived from patient records and may only be accessed and analyzed upon request and appropriate data use agreements with Vanderbilt University Medical Center. Requests to access the datasets should be directed to the corresponding author.

## Abbreviations

AE, adverse event; BMI, body mass index; CRP, C-reactive protein; DHEA, dehydroepiandrosterone; DHEA-S, dehydroepiandrosterone sulfate; EML, Essential Medicines List; FAI, free androgen index; HDL, high-density lipoprotein; HMG-CoA, β-Hydroxy β-methylglutaryl-CoA; HMGCR, 3-hydroxy-3-methylglutaryl-CoA reductase; HOMA-IR, homeostatic model assessment for insulin resistance; LDL, low-density lipoprotein; PCOS, polycystic ovary syndrome; PheWAS, phenome-wide association study; Remedi, Repurposing Medicines Internationally; SHBG, sex hormone binding globulin; SNP, single nucleotide polymorphism; sVCAM-1, soluble vascular cell adhesion molecule-1; T, testosterone.

## References

[B1] Almalki H. H. Alshibani T. M. Alhifany A. A. Almohammed O. A. (2020). Comparative efficacy of statins, metformin, spironolactone and combined oral contraceptives in reducing testosterone levels in women with polycystic ovary syndrome: a network meta-analysis of randomized clinical trials. BMC Womens Health 20 (1), 68. 10.1186/s12905-020-00919-5 32248801 PMC7132972

[B2] Ashraf S. Nabi M. Rasool S. ul A. Rashid F. Amin S. (2019). Hyperandrogenism in polycystic ovarian syndrome and role of CYP gene variants: a review. Egypt J. Med. Hum. Genet. 20 (1), 25. 10.1186/s43042-019-0031-4

[B3] Author anonymous (2025a). EMLs around the world. Available online at: https://global.essentialmeds.org/dashboard/medicines.

[B4] Author anonymous (2025b). 5-75356374-A-G | gnomAD v4.1.0 | gnomAD. Available online at: https://gnomad.broadinstitute.org/variant/5-75356374-A-G?dataset=gnomad_r4.

[B5] Azargoon A. Ghorbani R. Faraji Z. (2013). Effects of simvastatin pretreatment on clomiphene response in clomiphene - resistant women with polycystic ovary syndrome. J. Fam. Reprod. Health 7 (4), 165–170. PMC4064748 24971120

[B6] Banaszewska B. Pawelczyk L. Spaczynski R. Z. Dziura J. Duleba A. J. (2007). Effects of simvastatin and oral contraceptive agent on polycystic ovary syndrome: prospective, randomized, crossover trial. J. Clin. Endocrinol. Metab. 92 (2), 456–461. 10.1210/jc.2006-1988 17105841

[B7] Banaszewska B. Pawelczyk L. Spaczynski R. Z. Duleba A. J. (2009). Comparison of simvastatin and metformin in treatment of polycystic ovary syndrome: prospective randomized trial. J. Clin. Endocrinol. Metab. 94 (12), 4938–4945. 10.1210/jc.2009-1674 19890022 PMC2795658

[B8] Banaszewska B. Pawelczyk L. Spaczynski R. Z. Duleba A. J. (2011). Effects of simvastatin and metformin on polycystic ovary syndrome after six months of treatment. J. Clin. Endocrinol. Metab. 96 (11), 3493–3501. 10.1210/jc.2011-0501 21865358 PMC3205889

[B9] Baxi S. M. Beall R. Yang J. Mackey T. K. (2019). A multidisciplinary review of the policy, intellectual property rights, and international trade environment for access and affordability to essential cancer medications. Glob. Health 15, 57. 10.1186/s12992-019-0497-3 PMC6751842 31533850

[B10] Cassidy-Vu L. Joe E. Kirk J. K. (2016). Role of statin drugs for polycystic ovary syndrome. J. Fam. Reprod. Health 10 (4), 165–175. PMC5440815 28546815

[B11] Celik O. Acbay O. (2012). Effects of metformin plus rosuvastatin on hyperandrogenism in polycystic ovary syndrome patients with hyperlipidemia and impaired glucose tolerance. J. Endocrinol. Invest 35 (10), 905–910. 10.3275/8371 22522778

[B12] Challa A. P. Lavieri R. R. Lewis J. T. Zaleski N. M. Shirey-Rice J. K. Harris P. A. (2019). Systematically prioritizing candidates in genome-based drug repurposing. Assay. Drug Dev. Technol. 17 (8), 352–363. 10.1089/adt.2019.950 31769998 PMC6921094

[B13] Challa A. P. Zaleski N. M. Jerome R. N. Lavieri R. R. Shirey-Rice J. K. Barnado A. (2021). Human and machine intelligence together drive drug repurposing in rare diseases. Front. Genet. 12, 707836. 10.3389/fgene.2021.707836 34394194 PMC8355705

[B14] Chen L. L. Zheng J. H. (2021). Effects of atorvastatin on the insulin resistance in women of polycystic ovary syndrome: a systematic review and meta-analysis. Med. Baltim. 100 (24), e26289. 10.1097/MD.0000000000026289 PMC8213267 34128863

[B15] Chen J. Huang C. Zhang T. Gong W. Deng X. Liu H. (2021). The effects of statins on hyperandrogenism in women with polycystic ovary syndrome: a systematic review and meta-analysis of randomized controlled trials. Reprod. Biol. Endocrinol. 19 (1), 189. 10.1186/s12958-021-00863-5 34930305 PMC8686603

[B16] de Melo A. S. dos Reis R. M. Ferriani R. A. Vieira C. S. (2017). Hormonal contraception in women with polycystic ovary syndrome: choices, challenges, and noncontraceptive benefits. Open Access J. Contracept. 8, 13–23. 10.2147/OAJC.S85543 29386951 PMC5774551

[B17] Denny J. C. Ritchie M. D. Basford M. A. Pulley J. M. Bastarache L. Brown-Gentry K. (2010). PheWAS: demonstrating the feasibility of a phenome-wide scan to discover gene-disease associations. Bioinforma. Oxf Engl. 26 (9), 1205–1210. 10.1093/bioinformatics/btq126 PMC2859132 20335276

[B18] Denny J. C. Bastarache L. Ritchie M. D. Carroll R. J. Zink R. Mosley J. D. (2013). Systematic comparison of phenome-wide association study of electronic medical record data and genome-wide association study data. Nat. Biotechnol. 31 (12), 1102–1110. 10.1038/nbt.2749 24270849 PMC3969265

[B19] Deswal R. Narwal V. Dang A. Pundir C. S. (2020). The prevalence of polycystic ovary syndrome: a brief systematic review. J. Hum. Reprod. Sci. 13 (4), 261–271. 10.4103/jhrs.JHRS_95_18 33627974 PMC7879843

[B20] Di Stasi S. L. MacLeod T. D. Winters J. D. Binder-Macleod S. A. (2010). Effects of statins on skeletal muscle: a perspective for physical therapists. Phys. Ther. 90 (10), 1530–1542. 10.2522/ptj.20090251 20688875 PMC2949584

[B21] Duleba A. J. Banaszewska B. Spaczynski R. Z. Pawelczyk L. (2006). Simvastatin improves biochemical parameters in women with polycystic ovary syndrome: results of a prospective, randomized trial. Fertil. Steril. 85 (4), 996–1001. 10.1016/j.fertnstert.2005.09.030 16580386

[B22] Eacker S. M. Agrawal N. Qian K. Dichek H. L. Gong E. Y. Lee K. (2008). Hormonal regulation of testicular steroid and cholesterol homeostasis. Mol. Endocrinol. 22 (3), 623–635. 10.1210/me.2006-0534 18032697 PMC2262169

[B23] Franks S. Hardy K. (2018). Androgen action in the ovary. Front. Endocrinol. 9, 452. 10.3389/fendo.2018.00452 PMC6097027 30147675

[B24] Gao L. Zhao F. L. Li S. C. (2012). Statin is a reasonable treatment option for patients with Polycystic Ovary Syndrome: a meta-analysis of randomized controlled trials. Exp. Clin. Endocrinol. Diabetes Off. J. Ger. Soc. Endocrinol. Ger. Diabetes Assoc. 120 (6), 367–375. 10.1055/s-0032-1304619 22639397

[B25] Ghazeeri G. Abbas H. A. Skaff B. Harajly S. Awwad J. (2015). Inadequacy of initiating rosuvastatin then metformin on biochemical profile of polycystic ovarian syndrome patients. J. Endocrinol. Invest 38 (6), 643–651. 10.1007/s40618-015-0237-3 25722221

[B26] González-Castejón M. Marín F. Soler-Rivas C. Reglero G. Visioli F. Rodríguez-Casado A. (2011). Functional non-synonymous polymorphisms prediction methods: current approaches and future developments. Curr. Med. Chem. 18 (33), 5095–5103. 10.2174/092986711797636081 22050757

[B27] Hao S. L. Zhang C. L. Meng X. Y. (2023). Comparison of different drug for reducing testosterone levels in women with polycystic ovary syndrome: a systematic review and network meta-analysis. Med. Baltim. 102 (41), e35152. 10.1097/MD.0000000000035152 PMC10578672 37832133

[B28] Karakas S. E. Banaszewska B. Spaczynski R. Z. Pawelczyk L. Duleba A. (2013). Free fatty acid binding protein-4 and retinol binding protein-4 in polycystic ovary syndrome: response to simvastatin and metformin therapies. Gynecol. Endocrinol. Off. J. Int. Soc. Gynecol. Endocrinol. 29 (5), 483–487. 10.3109/09513590.2013.774360 23480783

[B29] Kaya C. Cengiz S. D. Berker B. Demirtaş S. Cesur M. Erdoğan G. (2009). Comparative effects of atorvastatin and simvastatin on the plasma total homocysteine levels in women with polycystic ovary syndrome: a prospective randomized study. Fertil. Steril. 92 (2), 635–642. 10.1016/j.fertnstert.2008.06.006 18692805

[B30] Kaya C. Pabuccu R. Cengiz S. D. Dünder I. (2010). Comparison of the effects of atorvastatin and simvastatin in women with polycystic ovary syndrome: a prospective, randomized study. Exp. Clin. Endocrinol. Diabetes Off. J. Ger. Soc. Endocrinol. Ger. Diabetes Assoc. 118 (3), 161–166. 10.1055/s-0029-1220770 20146169

[B31] Kazerooni T. Shojaei-Baghini A. Dehbashi S. Asadi N. Ghaffarpasand F. Kazerooni Y. (2010). Effects of metformin plus simvastatin on polycystic ovary syndrome: a prospective, randomized, double-blind, placebo-controlled study. Fertil. Steril. 94 (6), 2208–2213. 10.1016/j.fertnstert.2009.11.045 20079899

[B32] Khan M. J. Ullah A. Basit S. (2019). Genetic basis of polycystic ovary syndrome (PCOS): current perspectives. Appl. Clin. Genet. 12, 249–260. 10.2147/TACG.S200341 31920361 PMC6935309

[B33] Krysiak R. Okopien B. (2014). The effect of atorvastatin and atorvastatin-ezetimibe combination therapy on androgen production in hyperandrogenic women with elevated cholesterol levels. Exp. Clin. Endocrinol. Diabetes 123 (02), 75–79. 10.1055/s-0034-1394400 25350347

[B34] Krysiak R. Zmuda W. Okopien B. (2014). The effect of ezetimibe on androgen production in hypercholesterolemic women with polycystic ovary syndrome. Cardiovasc Ther. 32 (5), 219–223. 10.1111/1755-5922.12088 25056604

[B35] Laing R. Waning B. Gray A. Ford N.’t Hoen E. (2003). 25 years of the WHO essential medicines lists: progress and challenges. Lancet 361 (9370), 1723–1729. 10.1016/S0140-6736(03)13375-2 12767751

[B36] Legro R. S. Kunselman A. R. Dunaif A. (2001). Prevalence and predictors of dyslipidemia in women with polycystic ovary syndrome. Am. J. Med. 111 (8), 607–613. 10.1016/s0002-9343(01)00948-2 11755503

[B37] Liu Q. Xie Y. jie Qu L. hua Zhang M. xia cheng Mo Z. (2019). Dyslipidemia involvement in the development of polycystic ovary syndrome. Taiwan J. Obstet. Gynecol. 58 (4), 447–453. 10.1016/j.tjog.2019.05.003 31307731

[B38] Luotola K. Piltonen T. T. Puurunen J. Morin-Papunen L. C. Tapanainen J. S. (2018). Testosterone is associated with insulin resistance index independently of adiposity in women with polycystic ovary syndrome. Gynecol. Endocrinol. Off. J. Int. Soc. Gynecol. Endocrinol. 34 (1), 40–44. 10.1080/09513590.2017.1342793 28678568

[B39] Malik M. Tasnim N. Mahmud G. (2018). Effect of metformin alone compared with metformin plus simvastatin on polycystic ovarian syndrome in Pakistani women. J. Coll. Physicians Surg. Pak 28 (3), 184–187. 10.29271/jcpsp.2018.03.184 29544572

[B40] Mehrabian F. Ghasemi-Tehrani H. Mohamadkhani M. Moeinoddini M. Karimzadeh P. (2016). Comparison of the effects of metformin, flutamide plus oral contraceptives, and simvastatin on the metabolic consequences of polycystic ovary syndrome. J. Res. Med. Sci. Off. J. Isfahan Univ. Med. Sci. 21, 7. 10.4103/1735-1995.177354 PMC5122242 27904553

[B41] Melo A. S. de Dias S. V. Cavalli R. de C. Cardoso V. C. Bettiol H. Barbieri M. A. (2015). Pathogenesis of polycystic ovary syndrome: multifactorial assessment from the foetal stage to menopause. Reproduction 150 (1), R11–R24. 10.1530/REP-14-0499 25835506

[B42] Miao K. Zhou H. (2022). Effect of statins combined or not combined with metformin on polycystic ovary syndrome: a systematic review and meta-analysis. J. Obstet. Gynaecol. Res. 48, 1806–1815. 10.1111/jog.15301 35620917

[B43] Navali N. Pourabolghasem S. Fouladi R. F. Nikpour M. A. (2011). Therapeutic effects of biguanide vs. statin in polycystic ovary syndrome: a randomized clinical trial. Pak J. Biol. Sci. PJBS 14 (11), 658–663. 10.3923/pjbs.2011.658.663 22235508

[B44] Oguz S. H. Yildiz B. O. (2021). An update on contraception in polycystic ovary syndrome. Endocrinol. Metab. 36 (2), 296–311. 10.3803/EnM.2021.958 PMC8090477 33853290

[B45] Podfigurna-Stopa A. Luisi S. Regini C. Katulski K. Centini G. Meczekalski B. (2015). Mood disorders and quality of life in polycystic ovary syndrome. Gynecol. Endocrinol. Off. J. Int. Soc. Gynecol. Endocrinol. 31 (6), 431–434. 10.3109/09513590.2015.1009437 26204044

[B46] Pourmatroud E. Mohammadjafari R. Roozitalab M. (2015). Comparison of metformin and simvastatin administration in women with polycystic ovary syndrome before intra-cytoplasmic sperm injection cycle: a prospective, randomized, clinical trial study. Iran. Red. Crescent Med. J. 17 (12), e20082. 10.5812/ircmj.20082 26756007 PMC4706729

[B47] Pulley J. M. Shirey-Rice J. K. Lavieri R. R. Jerome R. N. Zaleski N. M. Aronoff D. M. (2017). Accelerating precision drug development and drug repurposing by leveraging human genetics. Assay. Drug Dev. Technol. 15 (3), 113–119. 10.1089/adt.2016.772 28379727 PMC5399743

[B48] Pulley J. M. Rhoads J. P. Jerome R. N. Challa A. P. Erreger K. B. Joly M. M. (2020). Using what we already have: uncovering new drug repurposing strategies in existing omics data. Annu. Rev. Pharmacol. Toxicol. 60, 333–352. 10.1146/annurev-pharmtox-010919-023537 31337270

[B49] Puurunen J. Piltonen T. Puukka K. Ruokonen A. Savolainen M. J. Bloigu R. (2013). Statin therapy worsens insulin sensitivity in women with polycystic ovary syndrome (PCOS): a prospective, randomized, double-blind, placebo-controlled study. J. Clin. Endocrinol. Metab. 98 (12), 4798–4807. 10.1210/jc.2013-2674 24152688

[B50] Raja-Khan N. Kunselman A. R. Hogeman C. S. Stetter C. M. Demers L. M. Legro R. S. (2011). Effects of atorvastatin on vascular function, inflammation, and androgens in women with polycystic ovary syndrome: a double-blind, randomized, placebo-controlled trial. Fertil. Steril. 95 (5), 1849–1852. 10.1016/j.fertnstert.2010.11.040 21144505 PMC3062732

[B51] Rashidi B. Abediasl J. Tehraninejad E. Rahmanpour H. Sills E. S. (2011). Simvastatin effects on androgens, inflammatory mediators, and endogenous pituitary gonadotropins among patients with PCOS undergoing IVF: results from a prospective, randomized, placebo-controlled clinical trial. J. Investig. Med. Off. Publ. Am. Fed. Clin. Res. 59 (6), 912–916. 10.2310/JIM.0b013e31821bfd9c 21527854

[B52] Reith C. Baigent C. Blackwell L. Emberson J. Spata E. Davies K. (2022). Effect of statin therapy on muscle symptoms: an individual participant data meta-analysis of large-scale, randomised, double-blind trials. Lancet 400 (10355), 832–845. 10.1016/s0140-6736(22)01545-8 36049498 PMC7613583

[B53] Sathyapalan T. Kilpatrick E. S. Coady A. M. Atkin S. L. (2009). The effect of atorvastatin in patients with polycystic ovary syndrome: a randomized double-blind placebo-controlled study. J. Clin. Endocrinol. Metab. 94 (1), 103–108. 10.1210/jc.2008-1750 18940877

[B54] Sathyapalan T. Shepherd J. Arnett C. Coady A. M. Kilpatrick E. S. Atkin S. L. (2010a). Atorvastatin increases 25-hydroxy vitamin D concentrations in patients with polycystic ovary syndrome. Clin. Chem. 56 (11), 1696–1700. 10.1373/clinchem.2010.144014 20817794

[B55] Sathyapalan T. Kilpatrick E. S. Coady A. M. Atkin S. L. (2010b). Atorvastatin pretreatment augments the effect of metformin in patients with polycystic ovary syndrome (PCOS). Clin. Endocrinol. (Oxf) 72 (4), 566–568. 10.1111/j.1365-2265.2009.03678.x 19681918

[B56] Sathyapalan T. Smith K. A. Coady A. M. Kilpatrick E. S. Atkin S. L. (2012a). Atorvastatin therapy decreases androstenedione and dehydroepiandrosterone sulphate concentrations in patients with polycystic ovary syndrome: randomized controlled study. Ann. Clin. Biochem. 49 (Pt 1), 80–85. 10.1258/acb.2011.011071 21972424

[B57] Sathyapalan T. Shepherd J. Coady A. M. Kilpatrick E. S. Atkin S. L. (2012b). Atorvastatin reduces malondialdehyde concentrations in patients with polycystic ovary syndrome. J. Clin. Endocrinol. Metab. 97 (11), 3951–3955. 10.1210/jc.2012-2279 22879630

[B58] Sathyapalan T. Coady A. M. Kilpatrick E. S. Atkin S. L. (2017). The effect of atorvastatin on pancreatic beta cell requirement in women with polycystic ovary syndrome. Endocr. Connect. 6 (8), 811–816. 10.1530/EC-17-0217 29018156 PMC5682417

[B59] Sathyapalan T. Hobkirk J. P. Javed Z. Carroll S. Coady A. M. Pemberton P. (2019). The effect of atorvastatin (and subsequent metformin) on adipose tissue acylation-stimulatory-protein concentration and inflammatory biomarkers in overweight/obese women with polycystic ovary syndrome. Front. Endocrinol. 10, 394. 10.3389/fendo.2019.00394 PMC6604602 31293514

[B60] Seyam E. Hefzy E. (2018). Long-term effects of combined simvastatin and metformin treatment on the clinical abnormalities and ovulation dysfunction in single young women with polycystic ovary syndrome. Gynecol. Endocrinol. Off. J. Int. Soc. Gynecol. Endocrinol. 34 (12), 1073–1080. 10.1080/09513590.2018.1490405 30044162

[B61] Seyam E. Al G. S. Abd Al G. A. Mohamed M. A. A. Youseff A. M. Ibrahim E. M. (2018). Evaluation of prolonged use of statins on the clinical and biochemical abnormalities and ovulation dysfunction in single young women with polycystic ovary syndrome. Gynecol. Endocrinol. Off. J. Int. Soc. Gynecol. Endocrinol. 34 (7), 589–596. 10.1080/09513590.2017.1418853 29258367

[B62] Shawish M. I. Bagheri B. Musini V. M. Adams S. P. Wright J. M. (2021). Effect of atorvastatin on testosterone levels. Cochrane Database Syst. Rev. 1 (1). 10.1002/14651858.CD013211.pub2 PMC8094971 33482034

[B63] Sicchia S. R. Maclean H. (2006). Globalization, poverty and women's health: mapping the connections. Can. J. Public Health Rev. Can. Santé Publique 97 (1), 69–71. 10.1007/BF03405219 PMC6976250 16512333

[B64] Skottheim I. B. Gedde-Dahl A. Hejazifar S. Hoel K. Asberg A. (2008). Statin induced myotoxicity: the lactone forms are more potent than the acid forms in human skeletal muscle cells *in vitro* . Eur. J. Pharm. Sci. Off. J. Eur. Fed. Pharm. Sci. 33 (4–5), 317–325. 10.1016/j.ejps.2007.12.009 18294823

[B65] Smals A. G. Weusten J. J. Benraad T. J. Kloppenborg P. W. (1991). The HMG-CoA reductase inhibitor simvastatin suppresses human testicular testosterone synthesis *in vitro* by a selective inhibitory effect on 17-ketosteroid-oxidoreductase enzyme activity. J. Steroid Biochem. Mol. Biol. 38 (4), 465–468. 10.1016/0960-0760(91)90333-z 2031860

[B66] Taylor and Francis (2020). Full article: quality of life and sexual function in women with polycystic ovary syndrome. Compr. Rev. 10.1080/09513590.2019.1670788

[B67] Thompson P. D. Clarkson P. Karas R. H. (2003). Statin-associated myopathy. JAMA 289 (13), 1681–1690. 10.1001/jama.289.13.1681 12672737

[B68] Vahedian-Azimi A. Makvandi S. Banach M. Reiner Ž. Sahebkar A. (2021). Fetal toxicity associated with statins: a systematic review and meta-analysis. Atherosclerosis 327, 59–67. 10.1016/j.atherosclerosis.2021.05.006 34044205

[B69] Wang F. F. Pan J. X. Wu Y. Zhu Y. H. Hardiman P. J. Qu F. (2018). American, European, and Chinese practice guidelines or consensuses of polycystic ovary syndrome: a comparative analysis. J. Zhejiang Univ. Sci. B 19 (5), 354–363. 10.1631/jzus.B1700074 29732746 PMC5962512

[B70] WHO (2025). WHO Infertility is a global public health issue. Available online at: http://www.who.int/reproductivehealth/topics/infertility/perspective/en/.

[B71] Wild R. A. Rizzo M. Clifton S. Carmina E. (2011). Lipid levels in polycystic ovary syndrome: systematic review and meta-analysis. Fertil. Steril. 95 (3), 1073–9.e11. 10.1016/j.fertnstert.2010.12.027 21247558

[B72] World Health Organization (2035). Women’s health. Available online at: https://www.who.int/health-topics/women-s-health.

[B73] Xiong T. Fraison E. Kolibianaki E. Costello M. F. Venetis C. Kostova E. B. (2023). Statins for women with polycystic ovary syndrome not actively trying to conceive. Cochrane Database Syst. Rev. 7 (7), CD008565. 10.1002/14651858.CD008565.pub3 37462232 PMC10353291

[B74] Yang B. Sun Z. J. Chen B. Zhang J. Zhao H. Li C. W. (2016). Statin ameliorates endothelial dysfunction and insulin resistance in Tibet women with polycystic ovary syndrome. Eur. Rev. Med. Pharmacol. Sci. 20 (6), 1185–1191. 27049276

[B75] Yang S. Gu Y. Y. Jing F. Yu C. X. Guan Q. B. (2019). The effect of statins on levels of dehydroepiandrosterone (DHEA) in women with polycystic ovary syndrome: a systematic review and meta-analysis. Med. Sci. Monit. Int. Med. J. Exp. Clin. Res. 25, 590–597. 10.12659/MSM.914128 PMC6348753 30698163

[B76] Yang X. Wu R. Qi D. Fu L. Song T. Wang Y. (2021). Profile of bile acid metabolomics in the follicular fluid of PCOS patients. Metabolites 11 (12), 845. 10.3390/metabo11120845 34940603 PMC8703527

[B77] Zarek J. Koren G. (2014). The fetal safety of statins: a systematic review and meta-analysis. J. Obstet. Gynaecol. Can. JOGC J. Obstet. Gynecol. Can. JOGC 36 (6), 506–509. 10.1016/S1701-2163(15)30565-X 24927189

[B78] ZOCOR (1991). Zocor package insert (Merck). Available online at: https://www.merck.com/product/usa/pi_circulars/z/zocor/zocor_pi.pdf.

